# Characteristics and Circumstances of U.S. Women Who Obtain Very Early and Second-Trimester Abortions

**DOI:** 10.1371/journal.pone.0169969

**Published:** 2017-01-25

**Authors:** Rachel K. Jones, Jenna Jerman

**Affiliations:** Research Division, Guttmacher Institute, New York, New York, United States of America; University of Ottawa, CANADA

## Abstract

**Objective:**

To determine which characteristics and circumstances were associated with very early and second-trimester abortion.

**Methods:**

Paper and pencil surveys were collected from a national sample of 8,380 non-hospital U.S. abortion patients in 2014 and 2015. We used self-reported LMP to calculate weeks gestation; when LMP was not provided we used self-reported weeks pregnant. We constructed two dependent variables: obtaining a very early abortion, defined as six weeks gestation or earlier, and obtaining second-trimester abortion, defined as occurring at 13 weeks gestation or later. We examined associations between the two measures of gestation and a range of characteristics and circumstances, including type of abortion waiting period in the patients’ state of residence.

**Results:**

Among first-trimester abortion patients, characteristics that decreased the likelihood of obtaining a very early abortion include being under the age of 20, relying on financial assistance to pay for the procedure, recent exposure to two or more disruptive events and living in a state that requires in-person counseling 24–72 hours prior to the procedure. Having a college degree and early recognition of pregnancy increased the likelihood of obtaining a very early abortion. Characteristics that increased the likelihood of obtaining a second-trimester abortion include being Black, having less than a high school degree, relying on financial assistance to pay for the procedure, living 25 or more miles from the facility and late recognition of pregnancy.

**Conclusions:**

While the availability of financial assistance may allow women to obtain abortions they would otherwise be unable to have, it may also result in delays in accessing care. If poor women had health insurance that covered abortion services, these delays could be alleviated. Since the study period, four additional states have started requiring that women obtain in-person counseling prior to obtaining an abortion, and the increase in these laws could slow down the trend in very early abortion.

## Introduction

For individuals who wish to terminate a pregnancy, timely access to abortion care is key. Access to abortion in the first trimester is particularly important, as second-trimester procedures are offered by fewer providers, are substantially more expensive [1] and introduce a slightly elevated risk of serious complications [2;3].

Since 1973, when abortion was legalized nationally, around 11% of abortions have occurred at or after 13 weeks gestation [4]. Prior research using data from patients suggests that several characteristics and circumstances increase the likelihood of obtaining a second-trimester abortion, including being a teen, being Black, using health insurance to pay for the procedure, difficulty finding a provider and, in particular, late recognition of pregnancy [5–7]. Several studies using aggregate state-level data also found that Mississippi’s in-person counseling requirement, implemented in 1992, was associated with a slight increase in second trimester abortions in the state [8–10]. Subsequent studies have not examined whether waiting periods were associated with an increase in second-trimester abortion in other states that have since implemented this type of waiting period.

While the proportion of abortions obtained in the second trimester has remained stable, the proportion of abortions that were very early, or performed at or before six weeks gestation, increased from 18% in 1997 [11] to 35% in 2012 [7]. This is likely due, at least in part, to increased reliance on manual vacuum aspiration (MVA) and early medication abortion (EMA) [12;13]. To date there is relatively little information about women who have very early abortions. Notably, abortions at six weeks gestation pose the same minimal risk of complications as abortions at 12 weeks gestation [3], and most first-trimester abortions typically cost the same regardless of gestation. In March 2016, the FDA increased the gestational limit for EMA from seven to 10 weeks, which means more women will have access to this option even if they experience delays in accessing services. Still, the majority of abortion patients, including 52% of those obtaining first-trimester abortions, would have preferred to have had their abortion earlier [14]. Additionally, EMA is slightly more successful the earlier in the pregnancy it is performed, and earlier gestational age is associated with decreased bleeding and spotting during EMA [15]. Very early abortion is widely available, and over 90% of non-hospital abortion-providing facilities offer abortions at five weeks’ gestation [1]. Understanding who is able to access abortion at very early gestations, and which factors are associated with a decreased likelihood of doing so, could potentially identify practices and policies to help more women access abortions earlier.

This study uses data from a national sample of 8,380 non-hospital U.S. abortion patients to determine which characteristics and circumstances were associated with obtaining very early and second-trimester abortions. During the study period, ten states required in-person counseling 24–72 hours prior to the abortion procedure, and an additional 14 states required counseling without the requirement of an in-person visit; we include a measure of type of waiting period to assess whether these regulations were associated with abortion timing.

## Materials and Methods

Data for the analyses come from the Guttmacher Institute’s 2014 Abortion Patient Survey (APS) [16;17]. The 2014 APS collected information from 8,380 abortion patients accessing services at 87 non-hospital facilities across the United States. Information was gathered using a four-page, paper-and-pencil, self-administered questionnaire, available in English or Spanish. Participating facilities were randomly selected, and the data are considered to be nationally representative of non-hospital abortion patients in the United States. The survey and data collection procedures were approved by the Guttmacher Institute’s Institutional Review Board. These data have been used in previously published analyses [16;17], but detailed information about the data collection techniques and weighting procedure is available as a supporting information file.

We examine two measures of gestation, both constructed from the same variable. Respondents were asked to provide the date the survey was administered and the date of their last menstrual period (LMP); these items were used to estimate gestation. For the 15% of respondents who did not provide an LMP, we used their answers on a follow up item asking “About how many weeks pregnant are you?” Our first dependent variable measures whether women were obtaining a very early abortion, defined as six weeks gestation or earlier [18;19]. We also assessed whether women were obtaining a second-trimester abortion, defined as abortions at 13 weeks or later. (Because most patients completed surveys with their intake forms prior to their procedure, it was not possible to determine if gestational age was confirmed via ultrasound.) Some 275 women (3% of the sample) did not answer either item (LMP or weeks pregnant) and were excluded from analyses.

The analyses include a number of independent variables. Basic demographic characteristics include age, relationship status at the time of conception (with cohabiting as a separate category), race and ethnicity and highest educational degree obtained. Our measure of fertility distinguishes between women who reported no prior pregnancies, only a prior birth, only a prior abortion or both. We adopted this strategy because it is possible that previously pregnant women would recognize the pregnancy sooner than those who had never been pregnant, and that those who had had a prior abortion would be able to find a provider sooner.

Situational characteristics used as independent variables included procedure payment type and exposure to disruptive life events. All respondents were asked how they were paying for the procedure, and we distinguished between the following payment methods: private insurance, Medicaid, financial assistance or clinic discount, self-pay and some other method of payment. Seven percent of respondents reported using more than one method of payment, most commonly financial assistance and self-pay. In instances where respondents reported multiple payment methods we prioritized based on the above list (e.g., private insurance was given priority over any other type of payment method reported). Four percent of respondents did not answer the item asking about method of payment, and rather than exclude them from the analyses, they were examined as a separate category.

Respondents were asked if they had experienced any of eight potentially disruptive events in the last 12 months including the death of a close friend or family member, falling behind on rent or mortgage, separating from a spouse or partner, being unemployed for a month or longer, having a dependent or family member with a serious health problem, having a baby, having a partner arrested or incarcerated and moving two or more times. We constructed a measure of cumulative disruptions according to whether respondents had experienced none, one, two or three or more events. We also included a measure of exposure to intimate partner violence (IPV) based on two items; the first asked whether the respondent had ever been physically abused by the man who got her pregnant, and the second asked whether he had ever forced her to do anything sexual she did not want to do.

Respondents were asked to provide the zip code for their current residence, and this information was used to estimate how far women lived from the facility where they obtained the abortion (based on Euclidean, or “straight line,” distance). Eight percent of women did not provide a zip code and are examined as a separate category. Six women who indicated they lived outside the United States were excluded from the analysis.

Pregnancy awareness was included as a control variable. All respondents were asked “About how many weeks pregnant where you when you found out you were pregnant?” We were unable to determine if women were reporting pregnancy awareness based on LMP or date of fertilization, and it is quite likely that many were reporting the latter (e.g., pregnancy awareness reported as six weeks was actually eight weeks LMP). Models looking at early abortion assessed whether the woman knew she was pregnant at or before the fourth week of pregnancy. We chose this cutoff because it was the earliest time at which a pregnancy could be confirmed (for women reporting based on LMP). Analyses that examine second-trimester abortion include a measure of whether the woman found out she was pregnant at seven weeks LMP or later. The cutoff of seven weeks was somewhat arbitrary, but was chosen because it was later than the average gestation at which most patients reported realizing they were pregnant (5 weeks) but was not so late that second-trimester abortion was the only option. Eight percent of respondents did not provide an answer to this question, and an additional 42 provided out of range responses. These respondents were coded to “0”—rather than to missing—which was the comparison group for each measure. We consider the pregnancy recognition variables to be crude measures both because of the relatively high level of missing values (coded to “0”) and because it is unclear if it was assessed based on LMP or fertilization.

Finally, we include a variable assessing whether and what type of waiting period was in place in the state the woman lived in: no waiting period, a counseling and waiting period that did not require an in-person visit, or a waiting period that did require an in-person visit.

In preliminary analyses we examined associations between the dependent variables and several other characteristics including poverty, number of prior births, pregnancy intention and contraceptive use. However, we determined that these measures were redundant with other variables or were not associated with either outcome, and they were not included in the current analyses.

We first examined the distribution of abortions by gestation. We next examined incidence of very early and second-trimester abortion according to each of the relevant independent variables. The 2014 APS included weights to adjust for non-response [16], and this was applied to all univariate and bivariate analyses. Multilevel mixed-effects logistic regression was used to estimate associations between the independent variables and the two outcomes. A mixed-effects model accounts for the hierarchical nature of the data, or the fact that patients were clustered within facilities. Since the models include a measure of state policies (waiting periods), it is particularly important to take this clustering into account. Analyses of early abortion were limited to those in the first trimester (12 weeks and earlier) so as to more clearly assess associations with this outcome apart from the influence of factors associated with obtaining an abortion in the second trimester.

To evaluate the robustness of the findings, we conducted several sensitivity analyses. We re-ran both multilevel models excluding women who did not provide an LMP date, as some women who answered the item about weeks pregnant may have reported weeks since fertilization (e.g., a pregnancy dated six weeks after LMP may have been reported as a four weeks since fertilization likely occurred two weeks after LMP). Additionally, most states with waiting periods also have other abortion restrictions that could potentially delay access to services. These include restricting private insurance coverage of abortion [20] and laws that single out abortion providers and require them to implement onerous regulations, typically referred to as the targeted regulation of abortion providers, or TRAP laws [21]. Thus we also examined models that included measures of whether women lived in a state with these two laws in addition to type of waiting period.

## Results

**Fig 1:**
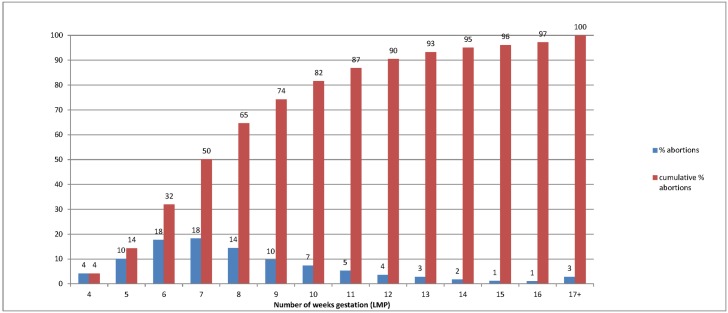
Percentage of abortions by weeks gestation (LMP) and cumulative percentage of abortions by gestation.

Only a very small proportion of non-hospital abortion patients, 4%, obtained their abortion at four weeks gestation or earlier (Fig 1). The proportion increased substantially in each subsequent week until the 11^th^ week of pregnancy. Ninety percent of the patients were in the first trimester, obtaining abortions at 12 weeks LMP or earlier, and, most commonly, 60% of women obtained abortions between five and eight weeks LMP.

The characteristics of women obtaining abortions have been described elsewhere [16], and we focus on those that are new to the current analyses (Table 1). Most commonly, 45% of patients paid out of pocket for abortion care, and Medicaid was the second most common method of payment. (The overwhelming majority of these patients lived in one of the 15 states that use their own Medicaid funds to cover abortion.) Women were about as likely to rely on financial assistance (13%) as they were to use private insurance to pay for the procedure (14%). Financial assistance refers to discounts provided by the clinic or subsidies available to qualified patients at some facilities; notably, slightly more than one-third of women relying on financial assistance, 5% of the sample, also reported paying at least some out-of-pocket costs (not shown).

**Table 1:** Characteristics of non-hospital U.S. abortions patients, 2014.

Patient characteristic	%	N
**Age**		
<15–17	3.6	288
18 19	8.2	659
20–24	33.6	2782
25–29	26.3	2154
30–34	16.0	1259
35+	12.2	956
**Union status**		
Married	14.4	1152
Cohabiting	31.0	2516
Never married	45.8	3704
Previously married	8.8	726
**Race and ethnicity**		
Asian Pacific Islander	4.7	374
Black	24.8	2021
White	39.0	3179
Other	2.5	207
Multiracial	4.5	374
Hispanic	24.5	1943
**Nativity**		
U.S.-born	84.0	6846
Foreign-born	16.0	1252
**Prior fertility**		
No prior pregnancies	29.2	2370
Prior birth(s) only	26.0	2132
Prior abortion(s) only	11.7	943
Prior birth and abortion	33.1	2653
**Education**		
Not a high school graduate	12.2	973
High school graduate or GED	29.0	2367
Some college or associates degree	39.2	3203
College graduate	19.7	1555
**Payment method**†		
Private insurance	14.1	1140
Medicaid	21.9	1779
Financial assistance	13.2	1066
Out of pocket	45.4	3680
Other	1.8	145
Missing	3.6	288
**Exposure to violence by man who impregnated respondent**		
No	95.4	7715
Yes	4.7	383
**Exposure to disruptive events in last 12 months**		
0	44.8	3607
1	32.2	2618
2	12.8	1040
3	10.2	833
**Distance from provider**		
<25 miles	66.7	5375
25–49 miles	12.6	1024
50–100 miles	8.0	668
>100 miles	5.1	429
missing	7.6	602
**When knew pregnant**		
≤ 4 weeks	36.3	2960
5 or 6 weeks	43.0	3,509
≥ 7 weeks	20.6	1629
**Waiting period**		
None	54.1	4304
Only waiting	21.7	1904
In-person visit required	24.2	1890

^†^Respondents could report more than one method of payment, and those reporting multiple methods were prioritized in this order (e.g., private insurance was given priority over all others).

Five percent of abortion patients had been exposed to IPV by the man who got them pregnant, and 55% had been exposed to one or more disruptive events in the last 12 months. Slightly more than one-third of abortion patients recognized the pregnancy at four weeks or earlier, though 21% did not realize they were pregnant until the seventh week or later. Among second-trimester abortion patients, 60% did not realize they were pregnant until the 7^th^ week or later (not shown). On average, women reported that they discovered the pregnancy during the fifth week, but second trimester patients discovered the pregnancy during the ninth week (not shown). Finally, 46% of patients lived in a state with a waiting period, including 24% who lived in states that had an in-person counseling requirement. Abortion patients in states with an in-person waiting period are likely overrepresented in the data. In 2011, the most recent year for which there are data, 36% of abortions occurred in states with a waiting period, including 18% in states with an in-person counseling requirement [22]. Sixty-five percent of abortions occurred in states with no waiting period, compared to 54% of the sample.

Among first-trimester abortion patients, a number of characteristics were associated with obtaining an abortion at six weeks LMP or earlier (Table 2). The proportion of patients obtaining very early abortions largely increased with age. In the multilevel mixed-effects model, both minors and older adolescents were less likely to be obtaining a very early abortion compared to patients aged 20–24 (OR .65, 95% CI .45-.93 and OR .79, 95% CI .63-.99). The proportion of abortions that were very early differed little between most education groups though the proportion was higher, 43%, among those with college degrees; this association was maintained in the multivariable analyses (OR 1.42, 95% CI 1.21–1.67). When examined according to method of payment, patients relying on financial assistance had the lowest incidence of very early abortion (24%). This negative association was maintained in the multivariable analyses when compared to women who paid out of pocket for their procedure (OR .73, 95% CI .60-.87). As exposure to disruptive events increased, the proportion of women obtaining a very early abortion decreased, and the multivariate analysis revealed that those exposed to two or more disruptive were less likely to obtain an early abortion relative to those who had no exposure.

**Table 2:** Frequency of very early abortion by patient characteristics and odds ratios from mixed-effects logistic regression models examining characteristics associated with very early abortions.

Patient characteristic	% < = 6 weeks	OR (95% CI)	P-value
**Total**	35.5		
**Age**			
<15–17	26.3	0.65 (0.45, 0.93)	.02
18 19	28.8	0.79 (0.63, 0.99)	.04
20–24	33.1	ref.	
25–29	38.0	1.20 (1.05, 1.38)	.01
30–34	35.9	0.97 (0.82, 1.15)	.73
35+	39.6	1.13 (0.93, 1.38)	.20
**Union status**			
Married	41.1	1.17 (0.98, 1.38)	.08
Cohabiting	32.7	0.93 (0.82, 1.05)	.23
Never married	34.2	ref.	
Previously married	37.2	1.07 (0.87, 1.30)	.54
**Race and ethnicity**			
Asian Pacific Islander	40.2	0.98 (0.75, 1.29)	.89
Black	32.4	0.93 (0.80, 1.08)	.36
White	35.0	ref.	
Other	36.1	1.17 (0.83, 1.65)	.37
Multiracial	31.3	0.82 (0.63, 1.07)	.15
Hispanic	37.2	1.03 (0.88, 1.20)	.75
**Nativity**			
U.S.-born	33.9	ref.	
Foreign-born	41.1	1.11 (0.94, 1.30)	.22
**Prior fertility**			
No prior pregnancies	35.3	ref.	
Prior birth(s) only	33.2	0.85 (0.72, 1.00)	.05
Prior abortion(s) only	36.6	0.98 (0.82, 1.18)	.84
Prior birth and abortion	35.8	0.97 (0.82, 1.14)	.67
**Education**			
Not a high school graduate	32.9	1.06 (0.86, 1.30)	.58
High school graduate or GED	31.7	ref.	
Some college or associates degree	33.9	1.10 (0.96, 1.25)	.16
College graduate	43.2	1.42 (1.21, 1.67)	<.001
**Payment method**†			
Private insurance	40.4	1.08 (0.92, 1.28)	.33
Medicaid	36.2	0.89 (0.75, 1.06)	.19
Financial assistance	24.4	0.73 (0.60, 0.87)	.00
Out of pocket	35.3	ref.	
Other	32.6	0.76 (0.50, 1.15)	.20
Missing	42.6	1.16 (0.87, 1.55)	.32
**Exposure to violence by man who impregnated respondent**			
No	35.3	ref.	
Yes	30.7	0.97 (0.74, 1.26)	.82
**Exposure to disruptive events in last 12 months**			
0	37.2	ref.	
1	35.9	0.99 (0.88, 1.12)	.90
2	29.9	0.80 (0.67, 0.95)	.01
3	28.9	0.76 (0.63, 0.93)	.01
**Distance from provider**			
<25 miles	36.7	ref.	
25–49 miles	32.8	0.87 (0.74, 1.03)	.10
50–100 miles	28.2	0.79 (0.64, 0.97)	.03
>100 miles	30.7	1.00 (0.77, 1.30)	.98
missing	33.7	0.92 (0.75, 1.13)	.44
**When knew pregnant**			
≤ 4 weeks	49.9	2.95 (2.66, 3.28)	<.001
>4 weeks	25.8	ref.	
**Waiting period**			
None	40.0	ref.	
Only waiting	34.7	0.85 (0.66, 1.09)	.21
In-person visit required	24.6	0.51 (0.39, 0.66)	<.001
**Intercept**	na	0.39 (0.29, 0.53)	<.001
**Number of respondents**	7,327		

OR = odds ratio; CI = confidence interval

^†^Respondents could report more than one method of payment, and those reporting multiple methods were prioritized in this order (e.g., private insurance was given priority over all others).

The proportion of first-trimester patients obtaining a very early procedures was greatest for those patients living closest to the facility (37%). When other factors were taken into account, women who lived 50–100 miles from the facility were the only ones less likely to be obtaining an early abortion compared to those who lived within 25 miles (OR .79, 95% CI .64-.97). Half of women who recognized the pregnancy at four weeks or earlier obtained a very early abortion, and even when other factors were taken into account they had nearly three times the odds of obtaining the abortion at six weeks gestation or earlier (OR 2.95, 95% CI 2.66–3.28).

While 40% of women who lived in states with no waiting period obtained a very early abortion, only 25% of those in states that required an in-person visit did so. This association was maintained in the mixed-effects model, and for women who lived in a state with an in-person counseling requirement the odds of obtaining a very early abortion were almost half those compared to women living in a state with no waiting period (OR .51, 95% CI .39-.66).

The sensitivity analyses suggest that most of the associations were robust (S1 Table). In the model that excluded the 1,016 women who did not provide an LMP, the association for 18–19 year olds remained negative but was no longer statistically significant (OR .82, 95% CI .64–1.04). However, this was the only association that was altered. The findings were unchanged in the model that included other state restrictions.

Many of the characteristics associated with very early abortion among first trimester patients had opposite associations with obtaining a second-trimester abortion (Table 3). Patients aged 18–19 had the highest proportion obtaining second-trimester procedures, and even when other factors were taken into account they were slightly more likely than patients aged 20–24 to have an abortion at 13 weeks gestation or later (OR 1.40, 95% CI 1.03–1.91). Black abortion patients had the highest proportion obtaining second-trimester procedures, and the multivariable analyses showed this group was slightly more likely than white patients to obtain an abortion in the second trimester (OR 1.50, 95% CI 1.18–1.90). Women born outside the United States were at decreased risk of obtaining an abortion at 13 weeks or later (OR .69, 95% CI .51-.93). As education increased, the proportion of abortions that were second-trimester procedures decreased, and these associations were maintained in the mixed-effects model.

**Table 3:** Frequency of second-trimester abortion by patient characteristics and odds ratios from mixed-effects logistic regression models examining characteristics associated with second-trimester abortions.

Patient characteristic	% >12 weeks	OR (95% CI)	P-value
**Total**	10.0		
**Age**			
<15–17	12.1	0.64 (0.39, 1.06)	.09
18 19	13.7	1.40 (1.03, 1.91)	.03
20–24	10.5	ref.	
25–29	9.2	0.86 (0.69, 1.08)	.21
30–34	8.9	0.93 (0.70, 1.23)	.61
35+	8.5	0.93 (0.67, 1.30)	.69
**Union status**			
Married	7.4	1.21 (0.89, 1.65)	.22
Cohabiting	12.1	1.37 (1.12, 1.66)	.00
Never married	9.7	ref.	
Previously married	8.0	1.04 (0.73, 1.49)	.82
**Race and ethnicity**			
Asian Pacific Islander	9.0	03 (0.63, 1.66)1.	.92
Black	13.2	1.50 (1.18, 1.90)	.00
White	8.5	ref.	
Other	12.0	1.12 (0.66, 1.90)	.69
Multiracial	11.2	1.11 (0.75, 1.66)	.60
Hispanic	8.8	0.96 (0.73, 1.24)	.73
**Nativity**			
U.S.-born	10.6	ref.	
Foreign-born	6.7	0.69 (0.51, 0.93)	.01
**Prior fertility**			
No prior pregnancies	8.9	ref.	
Prior birth(s) only	9.9	1.03 (0.79, 1.34)	.81
Prior abortion(s) only	8.3	0.90 (0.65, 1.24)	.52
Prior birth and abortion	11.5	1.11 (0.85, 1.45)	.45
**Education**			
Not a high school graduate	14.6	1.48 (1.12, 1.96)	.01
High school graduate or GED	11.7	ref.	
Some college or associates degree	9.0	0.72 (0.59, 0.89)	.00
College graduate	6.4	0.67 (0.50, 0.89)	.01
**Payment method**†			
Private insurance	8.7	1.13 (0.85, 1.51)	.40
Medicaid	13.2	1.18 (0.88, 1.58)	.28
Financial assistance	15.2	1.59 (1.24, 2.06)	<.001
Out of pocket	7.2	ref.	
Other	10.7	1.32 (0.71, 2.47)	.38
Missing	10.9	1.27 (0.80, 2.02)	.32
**Exposure to violence by man who impregnated respondent**			
No	9.7	ref.	
Yes	15.8	1.39 (0.98, 1.96)	.06
**Exposure to disruptive events in last 12 months**			
0	8.0	ref.	
1	10.1	1.06 (0.86, 1.29)	.59
2	12.3	1.17 (0.90, 1.53)	.23
3	15.0	1.32 (1.00, 1.75)	.05
**Distance from provider**			
<25 miles	8.8	ref.	
25–49 miles	10.6	1.24 (0.95, 1.62)	.11
50–100 miles	13.5	1.77 (1.31, 2.38)	<.001
>100 miles	17.3	2.06 (1.45, 2.93)	<.001
missing	10.7	1.27 (0.92, 1.76)	.14
**When knew pregnant**			
<7 weeks	4.9	ref.	
≥ 7 weeks	29.3	6.76 (5.69, 8.01)	<.001
**Waiting period**			
None	10.4	ref.	
Only waiting	10.0	1.01 (0.67, 1.53)	.95
In-person visit required	8.9	0.84 (0.55, 1.30)	.44
**Intercept**		0.04 (0.02, 0.07)	<.001
**Number of respondents**	8,099		

OR = odds ratio; CI = confidence interval

^†^Respondents could report more than one method of payment, and those reporting multiple methods were prioritized in this order (e.g., private insurance was given priority over all others).

Thirteen to 15% of women who were using financial assistance or Medicaid to pay for the procedure had an abortion at 13 weeks or later, and this was higher than for patients using other methods of payment (7–11%). However, in the mixed-effects model, only those relying on financial assistance were more likely than those paying out of pocket to be obtaining a second-trimester abortion (OR 1.59, 95% CI 1.24–2.06). The farther a patient lived from the facility, the more likely she was to be obtaining an abortion in the second trimester; the multivariable model showed that women who lived 50 or more miles from the facility were more likely than those who lived within 25 miles of it to be obtaining a second-trimester abortion. Among women who did not recognize they were pregnant until at least the seventh week of pregnancy, 29% were obtaining second trimester procedures, compared to 5% of those who recognized the pregnancy earlier. This association was also strong in the mixed-effects model (OR 6.76, 95% CI 5.69–8.01). While later recognition of pregnancy was associated with an increased likelihood of obtaining a second-trimester abortion, 61% of second-trimester patients recognized they were pregnant in the first trimester (not shown). Waiting period was not associated with second-trimester abortion.

The sensitivity analyses resulted in two substantive differences. In the model that excluded women who did not provide an LMP, women born outside the United States were no longer significantly less likely to obtain a second-trimester abortion, and women with college degrees no longer differed from those who graduated from high school (S2 Table). Associations in the model that included other state abortion restrictions were unchanged.

## Discussion

It follows that characteristics that increase the likelihood of obtaining a second-trimester abortion would decrease the likelihood of obtaining an early abortion, and several characteristics were associated with both of the outcomes examined in this study.

It continued to be the case that adolescents aged 18–19 were more likely than women aged 20–24 to be in the second-trimester when they obtained their abortions [6]. Both younger and older adolescents were less likely to obtain a very early abortion. Most young adults have never been pregnant, and it may take longer for them to recognize a pregnancy. Additionally, they may need more time to make a decision about the pregnancy [14].

The more education a woman had, the less likely she was to obtain an abortion in the second trimester, and having a college degree was associated with obtaining a very early abortion. Women with more education may have higher levels of health literacy, allowing them to recognize a pregnancy sooner and find an abortion provider more quickly.

In 2014, abortion patients were as likely to rely on financial assistance to pay for abortion services as they were to use private insurance. We found that reliance on financial assistance was associated with a decreased likelihood of very early abortion and an increased likelihood of second-trimester abortion. It is possible that the time it takes to apply for financial assistance can result in delays that reduce access to very early abortion. Prior research has documented that financial barriers result in delays accessing care, particularly among those obtaining second-trimester abortions [5;14;23–25]. In some cases, women who sought funding in the first trimester may have been in the second trimester by time funding was obtained [23;24]. Additionally, second-trimester abortions are substantially more expensive than first-trimester procedures [1], and financial assistance may be the only way some women can afford the abortion. If women who relied on financial assistance to pay for the procedure had health insurance that covered abortion care, these delays would likely be alleviated.

Women using private insurance to pay for the procedure were no more or less likely than those paying out of pocket to be obtaining an early or second-trimester abortion. While health insurance coverage presumably increases access to health care, abortion may be the exception. Most women with private health insurance pay out of pocket for abortion care [16], and self-paying patients may be similar to those using their health insurance (especially once reliance on financial assistance is taken into account). Women with private insurance coverage may have paid out of pocket because they had high deductibles, their plans did not cover abortion care, or because they did not want the abortion on their health insurance records [26].

Timing of pregnancy recognition was the factor most strongly associated with obtaining a very early or second-trimester abortion. We included this measure as a control variable; for example, women who do not realize they are pregnant before the sixth week of pregnancy are not eligible to obtain a very early abortion. The strength of the association, particularly for second-trimester patients, could be interpreted to suggest that helping women recognize their pregnancies at an earlier stage could help sustain the increase in very early abortions and reduce the need for second-trimester procedures. At the same time, women who had previously been pregnant, and presumably knew the signs of pregnancy, were no more or less likely to be obtaining a very early or second-trimester abortion. Additionally, the majority of second-trimester abortion patients recognized the pregnancy in the first trimester and earlier recognition alone will not eliminate the need for these services.

Several characteristics were only associated with one outcome but not the other. Relative to white women, black women were more likely to obtain abortions in the second trimester. This association has been found in prior research [6;7], and it is possible that factors apart from education or pregnancy recognition result in delays in accessing care for this population. For example, at least one prior study found that Black and Hispanic women took a longer time to decide to have an abortion relative to white women [14].

Women who lived 50 or more miles from the facility where they obtained the abortion were more likely to be seeking second-trimester procedures compared to those who lived within 25 miles. It is possible that women who lived further from an abortion provider needed more time to find the facility and make arrangements, resulting in delays in accessing care. Alternately, second-trimester abortion services are offered by fewer facilities [1] and women may need to travel further to access them. This is particularly true for women who were obtaining abortions after the 14^th^ week of pregnancy as the number of non-hospital facilities offering services at later gestations declines somewhat dramatically [1].

Women who lived in states that required an in-person counseling visit 24–72 hours prior to the procedure were less likely to obtain an early abortion. Prior research suggests that in-person counseling requirements can result in delays longer than the 24–72 hours imposed by the laws [8;10;27]. For example, some facilities are only provide abortion care a few days, or even one day, per week. In states with an in-person counseling requirement this could hinder access to very early abortion.

This study has several limitations. Our measure of gestation was based on self-reported information and not on ultrasound. While gestational ages based on women’s reports of LMP are usually comparable to those based on ultrasound, when they are inaccurate they tend to underestimate gestation [28;29]. Women living in states with a waiting period were likely overrepresented in the data, and this may have given this particular characteristic more weight, or power, in the analyses. Hospitals were excluded from the study. Since a disproportionate share of abortions performed in hospitals are second-trimester procedures [30;31], this could also bias the results for analyses where second-trimester abortion was the dependent variable. Finally, while a number of characteristics were associated with both early and second-trimester abortion, it is worth noting that most of the associations were not particularly strong, insofar as most of the odds ratios were less than 2.00 or greater than .50.

## Conclusions

Our findings have two policy implications. Insurance coverage of abortion is subject to a number of regulations. For example, the Hyde Amendment bans abortion coverage through federal Medicaid except in cases of rape, incest or life endangerment. One consequence of this restriction is that many poor and low-income abortion patients have to rely on financial assistance to pay for the procedure. The availability of discounts and subsidies to cover some or all of this cost may make the procedure accessible to women who otherwise could not afford it, but it potentially results in delays in accessing care. In 2014, abortion patients who lived in states where Medicaid does not cover abortion were six times more likely to rely on financial assistance to pay for abortion care than patients in states where abortion is covered [16]. If restrictions on insurance coverage of abortion—particularly under Medicaid—were removed, it could facilitate access to early abortion and potentially decrease the need for second-trimester abortion. The findings also suggest that in-person counseling requirements can reduce access to very early abortion. During the study period, ten states enforced this regulation, but since that time four more states have enacted this type of waiting period. If states continue to implement this restriction, it could diminish the trend in very early abortion.

## Supporting Information

S1 File Data collection for the 2014 U.S. Abortion Patient Survey. (DOCX)

S1 Table Odds ratios from mixed-effects logistic regression models examining characteristics associated with very early abortions (sensitivity analyses). (DOCX)

S2 Table Odds ratios from mixed-effects logistic regression models examining characteristics associated with second-trimester abortion (sensitivity analyses). (DOCX)
