# Supplementary material for: Characteristics and Circumstances of U.S. Women Who Obtain Very Early and Second-Trimester Abortions
Source: PLoS One. 2017 Jan 25;12(1):e0169969. doi: 10.1371/journal.pone.0169969 (PMC5266268; doi:10.1371/journal.pone.0169969)
Supplement: S1 File — (DOCX) [file pone.0169969.s001.docx]

**S1 File. Data collection for the 2014 U.S. Abortion Patient Survey**

Data for these analyses come from the Guttmacher Institute’s 2014 Abortion Patient Survey (APS). The 2014 APS collected information from 8,380 abortion patients accessing services at 87 non-hospital facilities across the United States. Information was gathered using a four-page, paper-and-pencil, self-administered questionnaire, available in English or Spanish. Participating facilities were randomly selected, and the data are considered to be nationally representative of non-hospital abortion patients in the United States. The survey and data collection procedures were approved by the Guttmacher Institute’s Institutional Review Board.

Participating facilities were sampled from the universe of all known non-hospital abortion-providing facilities as of 2011, according to information obtained from the Guttmacher Institute’s 2011 Abortion Provider Census [1]. Hospitals were excluded from the sampling universe because of the logistical difficulties with recruitment, and facilities with small caseloads were excluded because of the high likelihood that they would not provide any abortions during the survey period. Combined, these two types of facilities accounted for just 4% of all abortions in 2011 [1].

The universe was stratified by annual caseload of abortions (30–399; 400–1,999; 2,000–4,999; and 5,000 or more), and by whether they were affiliated with national organizations for women’s reproductive health (e.g., the National Abortion Federation). Within each stratum, facilities were ordered by census region and state. Facilities from each stratum were systematically sampled, and facilities with the largest caseloads were oversampled to ensure a diverse representation of facility types within the sample.

Selected facilities were then recruited and assigned to a survey period that was inversely proportional to the probability of being selected, ranging from two weeks to 12 weeks. Our goal was to recruit 113 facilities; the final sample was obtained from patients at 87 facilities (77% of the original goal). The most difficult providers to recruit and retain were those with the smallest caseload (30-399) and that were not affiliated with a national organization, as they often had small offices with limited staff. Common reasons facility contacts gave for declining to participate included limited staffing resources, concerns that their facility would not see any abortion patients during the study period, a belief that their patients would not be interested in participating and the research team’s inability to reach the appropriate staff member at the facility to discuss the study.

The questionnaire was distributed to patients during their clinic visit at the time facility staff determined was most appropriate. Communications with facility staff suggest that questionnaires were typically distributed with office or clinic intake forms. The introductory language on the front of the survey described the purpose of the study, indicated that the questionnaire was voluntary and anonymous and served as implied consent. All respondents were provided with an envelope in which to place the survey before they returned it to facility staff. At the end of each week, staff mailed survey packets back to the Institute. Between April 2014 and June 2015, participating facilities reported providing a total of 11,024 abortions. Surveys were considered usable if a respondent answered three of the four demographic items (age, ethnicity, race and type of insurance) at the very beginning of the survey (i.e., questions #1-#4) as well as at least one contraceptive item and one “personal information” item (e.g., education, marital status, etc.). Usable surveys were returned from 8,380 patients, for a response rate of 76%. For patients who refused or were unable to participate, an employee of the facility was asked to complete and return a small portion of the questionnaire covering the patient’s age, race and ethnicity and insurance coverage. This enabled us to assess whether patients who were missed differed from patients who completed the survey. Facility staff returned 1,066 surveys with basic demographic information; no information was available for the remaining 1,578 patients who declined to participate.

An estimated 1,800 additional surveys would have been obtained if all facilities that were approached had agreed to participate, and a three-stage weighting process was used to correct for any bias produced by deviation from the original sampling plan and patient nonresponse. Individual weights were developed to adjust for the demographic characteristics of the 1,066 nonrespondents for whom facility staff provided information. Next, facility-level weights adjusted for the other 1,578 nonrespondents for whom no demographic data were available. Third, stratum weights were constructed to correct for departures from the number of facilities to be sampled in each grouping by caseload and provider type.

Missing information on core demographic items was imputed using “hot-deck” single imputation. This method identifies variables most strongly associated with each item requiring imputation, and sorts the data file accordingly to replace the missing value with that from a similar, adjacent case.

Reference List

[1] Jones R, Jerman J. Abortion incidence and service availability in the United States, 2011. Perspectives on Sexual and Reproductive Health 2014;46(1):3-14.
