# Supplementary material for: Characteristics and Circumstances of U.S. Women Who Obtain Very Early and Second-Trimester Abortions
Source: PLoS One. 2017 Jan 25;12(1):e0169969. doi: 10.1371/journal.pone.0169969 (PMC5266268; doi:10.1371/journal.pone.0169969)
Supplement: S1 Table — (DOCX) [file pone.0169969.s002.docx]

**S1 Table. Odds ratios from mixed-effects logistic regression models examining characteristics associated with very early abortions (sensitivity analyses)**

|  | | | | |
| --- | --- | --- | --- | --- |
| **Patient characteristic** | **Model 1** | | **Model 2** | |
|  | **Excludes those missing LMP** | | **Includes other legal restrictions** | |
|  | **OR (95% CI)** | **P-value** | **OR (95% CI)** | **P-value** |
| **Age** |  |  |  |  |
| <15-17 | 0.62 (0.42, 0.92) | .02 | 0.65 (0.45, 0.93) | .02 |
| 18 19 | 0.82 (0.64, 1.04) | .10 | 0.79 (0.63, 0.99) | .04 |
| 20-24 | ref. |  | ref. |  |
| 25-29 | 1.23 (1.06, 1.43) | .01 | 1.20 (1.05, 1.38) | .01 |
| 30-34 | 0.98 (0.82, 1.17) | .82 | 0.97 (0.82, 1.15) | .74 |
| 35+ | 1.16 (0.94, 1.42) | .16 | 1.14 (0.94, 1.38) | .19 |
| **Union status** |  |  |  |  |
| Married | 1.19 (1.00, 1.43) | .06 | 1.16 (0.98, 1.38) | .09 |
| Cohabiting | 0.93 (0.82, 1.07) | .32 | 0.93 (0.82, 1.05) | .23 |
| Never married | ref. |  | ref. |  |
| Previously married | 1.13 (0.91, 1.39) | .26 | 1.06 (0.87, 1.30) | .55 |
| **Race and ethnicity** |  |  |  |  |
| Asian Pacific Islander | 1.12 (0.84, 1.49) | .45 | 0.99 (0.75, 1.29) | .92 |
| Black | 0.94 (0.80, 1.10) | .44 | 0.93 (0.80, 1.08) | .35 |
| White | ref. |  | ref. |  |
| Other | 1.13 (0.78, 1.62) | .52 | 1.17 (0.83, 1.65) | .37 |
| Multiracial | 0.84 (0.64, 1.11) | .23 | 0.82 (0.63, 1.07) | .15 |
| Hispanic | 1.04 (0.88, 1.22) | .67 | 1.03 (0.88, 1.20) | .72 |
| **Nativity** |  |  |  |  |
| U.S.-born | ref. |  | ref. |  |
| Foreign-born | 1.11 (0.94, 1.32) | .23 | 1.11 (0.94, 1.30) | .22 |
| **Prior fertility** |  |  |  |  |
| No prior pregnancies | ref. |  | ref. |  |
| Prior birth(s) only | 0.88 (0.74, 1.05) | .16 | 0.85 (0.73, 1.00) | .05 |
| Prior abortion(s) only | 1.03 (0.85, 1.25) | .78 | 0.98 (0.82, 1.18) | .84 |
| Prior birth and abortion | 0.97 (0.82, 1.16) | .76 | 0.96 (0.82, 1.14) | .67 |
| **Education** |  |  |  |  |
| Not a high school graduate | 1.09 (0.87, 1.36) | .47 | 1.06 (0.86, 1.31) | .56 |
| High school graduate or GED | ref. |  | ref. |  |
| Some college or associates degree | 1.08 (0.94, 1.24) | .28 | 1.10 (0.96, 1.26) | .15 |
| College graduate | 1.42 (1.19, 1.68) | <.001 | 1.42 (1.21, 1.67) | <.001 |
| **Payment method†** |  |  |  |  |
| Private insurance | 1.11 (0.93, 1.31) | .25 | 1.09 (0.93, 1.28) | .30 |
| Medicaid | 0.89 (0.74, 1.07) | .20 | 0.90 (0.76, 1.08) | .26 |
| Financial assistance | 0.76 (0.63, 0.93) | .01 | 0.72 (0.60, 0.87) | .00 |
| Out of pocket | ref. |  | ref. |  |
| Other | 0.62 (0.39, 0.99) | .05 | 0.77 (0.51, 1.16) | .21 |
| Missing | 1.08 (0.80, 1.47) | .61 | 1.17 (0.87, 1.57) | .29 |
| **Exposure to violence by man who impregnated respondent** | | |  |  |
| No | ref. |  | ref. |  |
| Yes | 1.06 (0.81, 1.41) | .66 | 0.97 (0.74, 1.26) | .80 |
| **Exposure to disruptive events in last 12 months** | |  |  |  |
| 0 | ref. |  | ref. |  |
| 1 | 0.97 (0.86, 1.11) | .69 | 0.99 (0.88, 1.12) | .90 |
| 2 | 0.79 (0.65, 0.94) | .01 | 0.80 (0.67, 0.95) | .01 |
| 3 | 0.74 (0.60, 0.92) | .01 | 0.76 (0.63, 0.93) | .01 |
| **Distance from provider** |  |  |  |  |
| <25 miles | ref. |  | ref. |  |
| 25-49 miles | 0.86 (0.72, 1.03) | .10 | 0.87 (0.74, 1.03) | .10 |
| 50-100 miles | 0.78 (0.62, 0.98) | .03 | 0.79 (0.64, 0.98) | .03 |
| >100 miles | 1.04 (0.79, 1.37) | .79 | 1.00 (0.77, 1.30) | .99 |
| missing | 0.90 (0.72, 1.13) | .36 | 0.92 (0.75, 1.13) | .45 |
| **When knew pregnant** |  |  |  |  |
| ≤ 4 weeks | 2.70 (2.41, 3.01) | <.001 | 2.95 (2.65, 3.27) | <.001 |
| >4 weeks | ref. |  | ref. |  |
| **Waiting period** |  |  |  |  |
| None | ref. |  | ref. |  |
| Only waiting | 0.87 (0.67, 1.13) | .30 | 0.78 (0.58, 1.05) | .10 |
| In-person visit required | 0.50 (0.38, 0.65) | <.001 | 0.45 (0.34, 0.61) | <.001 |
| **TRAP law** |  |  |  |  |
| No | na |  | ref. |  |
| Yes | na |  | 1.19 (0.95, 1.50) | .14 |
| **Restrictions on private ins. coverage of abortion** | |  |  |  |
| No | na |  | ref. |  |
| Yes | na |  | 1.04 (0.73, 1.46) | .84 |
| **Intercept** | 0.39 (0.29, 0.53) | <.001 | 0.37 (0.28, 0.50) | <.001 |
| **Number of respondents** | 6, 468 |  | 7,327 |  |

OR = odds ratio; CI = confidence interval

†Respondents could report more than one method of payment, and those reporting multiple methods were prioritized in this order (e.g., private insurance was given priority over all others)
