# Supplementary material for: Characteristics and Circumstances of U.S. Women Who Obtain Very Early and Second-Trimester Abortions
Source: PLoS One. 2017 Jan 25;12(1):e0169969. doi: 10.1371/journal.pone.0169969 (PMC5266268; doi:10.1371/journal.pone.0169969)
Supplement: S2 Table — (DOCX) [file pone.0169969.s003.docx]

**S2 Table. Odds ratios from mixed-effects logistic regression models examining characteristics associated with second-trimester abortion (sensitivity analyses)**

|  | | | | |
| --- | --- | --- | --- | --- |
| **Patient characteristic** | **Model 1** | | **Model 2** | |
|  | **Excludes those missing LMP** | | **Includes other legal restrictions** | |
|  | **OR (95% CI)** | **P-value** | **OR (95% CI)** | **P-value** |
| **Age** |  |  |  |  |
| <15-17 | 0.61 (0.34, 1.08) | .09 | 0.64 (0.39, 1.06) | .09 |
| 18 19 | 1.53 (1.07, 2.18) | .02 | 1.41 (1.03, 1.92) | .03 |
| 20-24 | ref. |  | ref. |  |
| 25-29 | 0.82 (0.63, 1.05) | .11 | 0.86 (0.69, 1.08) | .21 |
| 30-34 | 0.86 (0.63, 1.17) | .33 | 0.93 (0.70, 1.23) | .60 |
| 35+ | 0.90 (0.64, 1.28) | .57 | 0.94 (0.68, 1.30) | .70 |
| **Union status** |  |  |  |  |
| Married | 1.25 (0.89, 1.76) | .19 | 1.21 (0.89, 1.66) | .22 |
| Cohabiting | 1.39 (1.12, 1.72) | .00 | 1.37 (1.13, 1.66) | .00 |
| Never married | ref. |  | ref. |  |
| Previously married | 1.06 (0.72, 1.56) | .77 | 1.04 (0.73, 1.49) | .83 |
| **Race and ethnicity** |  |  |  |  |
| Asian Pacific Islander | 1.09 (0.65, 1.82) | .75 | 1.03 (0.64, 1.67) | .90 |
| Black | 1.59 (1.23, 2.05) | <.001 | 1.49 (1.17, 1.88) | .00 |
| White | ref. |  | ref. |  |
| Other | 1.18 (0.67, 2.07) | .56 | 1.11 (0.65, 1.89) | .70 |
| Multiracial | 1.04 (0.66, 1.64) | .85 | 1.11 (0.74, 1.66) | .60 |
| Hispanic | 0.91 (0.68, 1.21) | .51 | 0.96 (0.73, 1.24) | .73 |
| **Nativity** |  |  |  |  |
| U.S.-born | ref. |  | ref. |  |
| Foreign-born | 0.77 (0.56, 1.07) | .12 | 0.69 (0.51, 0.93) | .01 |
| **Prior fertility** |  |  |  |  |
| No prior pregnancies | ref. |  | ref. |  |
| Prior birth(s) only | 1.06 (0.79, 1.43) | .71 | 1.03 (0.79, 1.34) | .81 |
| Prior abortion(s) only | 0.95 (0.67, 1.37) | .80 | 0.90 (0.65, 1.24) | .52 |
| Prior birth and abortion | 1.20 (0.89, 1.61) | .24 | 1.11 (0.85, 1.44) | .46 |
| **Education** |  |  |  |  |
| Not a high school graduate | 1.61 (1.17, 2.20) | .00 | 1.48 (1.12, 1.96) | .01 |
| High school graduate or GED | ref. |  | ref. |  |
| Some college or associates deg | 0.78 (0.62, 0.98) | .03 | 0.72 (0.59, 0.89) | .00 |
| College graduate | 0.79 (0.58, 1.08) | .15 | 0.67 (0.50, 0.89) | .01 |
| **Payment method†** |  |  |  |  |
| Private insurance | 1.27 (0.93, 1.75) | .13 | 1.14 (0.86, 1.53) | .37 |
| Medicaid | 1.24 (0.89, 1.71) | .20 | 1.21 (0.90, 1.64) | .21 |
| Financial assistance | 1.70 (1.28, 2.25) | <.001 | 1.59 (1.23, 2.06) | <.001 |
| Out of pocket | ref. |  | ref. |  |
| Other | 1.53 (0.79, 2.98) | .21 | 1.34 (0.72, 2.50) | .36 |
| Missing | 1.53 (0.94, 2.50) | .09 | 1.29 (0.81, 2.06) | .28 |
| **Exposure to violence by man who impregnated respondent** | | |  |  |
| No | ref. |  | ref. |  |
| Yes | 1.13 (0.76, 1.70) | .54 | 1.39 (0.98, 1.96) | .06 |
| **Exposure to disruptive events in last 12 months** | |  |  |  |
| 0 | ref. |  | ref. |  |
| 1 | 1.02 (0.82, 1.28) | .85 | 1.06 (0.87, 1.30) | .57 |
| 2 | 1.13 (0.85, 1.51) | .41 | 1.18 (0.90, 1.53) | .23 |
| 3 | 1.29 (0.95, 1.76) | .11 | 1.33 (1.00, 1.76) | .05 |
| **Distance from provider** |  |  |  |  |
| <25 miles | ref. |  | ref. |  |
| 25-49 miles | 1.10 (0.82, 1.48) | .51 | 1.25 (0.96, 1.62) | .10 |
| 50-100 miles | 1.86 (1.34, 2.58) | <.001 | 1.77 (1.31, 2.38) | <.001 |
| >100 miles | 2.07 (1.40, 3.06) | <.001 | 2.05 (1.44, 2.92) | <.001 |
| missing | 1.20 (0.84, 1.74) | .32 | 1.28 (0.93, 1.77) | .14 |
| **When knew pregnant** |  |  |  |  |
| <7 weeks | ref. |  | ref. |  |
| ≥ 7 weeks | 6.85 (5.68, 8.28) | <.001 | 6.75 (5.69, 8.01) | <.001 |
| **Waiting period** |  |  |  |  |
| None | ref. |  | ref. |  |
| Only waiting | 0.99 (0.66, 1.49) | .96 | 0.96 (0.59, 1.55) | .86 |
| In-person visit required | 0.81 (0.52, 1.25) | .34 | 0.71 (0.43, 1.18) | .19 |
| **TRAP law** |  |  |  |  |
| No | na |  | ref. |  |
| Yes | na |  | 1.32 (0.88, 1.99) | .18 |
| **Restrictions on private ins. coverage of abortion** | |  |  |  |
| No |  |  | ref. |  |
| Yes |  |  | 0.85 (0.49, 1.48) | .57 |
| **Intercept** | 0.03 (0.02, 0.05) | <.001 | 0.04 (0.02, 0.06) | <.001 |
| **Number of respondents** | 7,083 |  | 8,099 |  |

OR = odds ratio; CI = confidence interval

†Respondents could report more than one method of payment, and those reporting multiple methods were prioritized in this order (e.g., private insurance was given priority over all others)
